# Knowledge, Intention, and Self-Efficacy Associated with Breastfeeding: Impact of These Factors on Breastfeeding during Postpartum Hospital Stays in Taiwanese Women

**DOI:** 10.3390/ijerph18095009

**Published:** 2021-05-09

**Authors:** Shu-Fang Vivienne Wu, Shu-Ching Chen, Hsiao-Yun Liu, Hsiu-Lan Lee, Yueh-E Lin

**Affiliations:** 1 School of Nursing, National Taipei University of Nursing and Health Sciences, Taipei 112, Taiwan; shufang@ntunhs.edu.tw; 2 School of Nursing and Geriatric and Long-Term Care Research Center, College of Nursing, Chang Gung University of Science and Technology, Taoyuan 333, Taiwan; shuching@gw.cgust.edu.tw; 3 Department of Radiation Oncology and Proton and Radiation Therapy Center, Chang Gung Memorial Hospital at Linkou, Taoyuan 333, Taiwan; 4 School of Nursing, College of Medicine, Chang Gung University, Taoyuan 333, Taiwan; 5 Department of Nursing, Chang Gung Memorial Hospital at Linkou, Taoyuan 333, Taiwan; yunn@cgmh.org.tw (H.-Y.L.); f22023@cgmh.org.tw (H.-L.L.); 6 Department of Nursing, College of Nursing, Chang Gung University of Science and Technology, Taoyuan 333, Taiwan

**Keywords:** breastfeeding, early postpartum, hospital stay, intention, knowledge, self-efficacy

## Abstract

Breastfeeding knowledge, intention, and self-efficacy affect breastfeeding rates during the postpartum period. Insufficient knowledge, lack of intention, and poor breastfeeding self-efficacy reduce the likelihood of breastfeeding postpartum. The purposes of this study were to (1) assess women’s intention to breastfeed and knowledge and self-efficacy regarding breastfeeding following childbirth, and to (2) identify the factors associated with postpartum breastfeeding during women’s hospital stays. This longitudinal study with a pretest and posttest design study recruited pregnant women from the gynecology and obstetrics outpatient departments and inpatient wards at a medical center in northern Taiwan. Demographic and obstetric characteristics were recorded, and participants were assessed using the Numeric Rating Scale, the Breastfeeding Knowledge Questionnaire, the Breastfeeding Self-Efficacy Scale—Short Form, and breastfeeding status postpartum. Of the 120 participants, 25% reported breastfeeding during the postpartum hospital stay. Postpartum breastfeeding was associated with lower levels of education and higher prenatal levels of breastfeeding intention. Establishing a breastfeeding-friendly environment in the family and workplace may effectively increase continued breastfeeding.

## 1. Introduction

Breastfeeding is defined as feeding infants breast milk exclusively, either directly from the breast or indirectly through expressed breast milk [1]. Globally, the rate of continued breastfeeding is more than 70% within one year postpartum [2]. In Taiwan, the breastfeeding rates were 66% during the hospital stay [3], 61.8% at 1 month postpartum [4], 44% at 3 months postpartum [5], and 24.3% at 6 months postpartum [6]. Breast milk contains immunoglobulins, hormones, enzymes, and growth factors [7], these components help improve infant immune response against viruses and bacteria [8,9,10], promote sensory and cognitive development [8,9], protect against allergies [8,9], protect against risk factors of chronic disease [8,9], increase intelligence, and reductions in overweight and diabetes [10]. The World Health Organization [11] and United Nations Children’s Fund [12] promote breast milk as the ideal food for infants, and recommend breastfeeding for the first six months to achieve optimal growth, development, and health. Limited knowledge about breastfeeding [13], low levels of breastfeeding intention [13], and poor breastfeeding self-efficacy [14,15] are associated with a lower likelihood of breastfeeding.

In one study [13], breastfeeding knowledge was quantified on the basis of mothers’ knowledge of specific aspects of breastfeeding, including benefit, barriers, and notes of breastfeeding [13]. The results suggested that women living without relatives and those who were not offered readymade liquid formula in the hospital had increased levels of postpartum breastfeeding [13]. Previous studies have also observed higher rates of postpartum breastfeeding in women who had better breastfeeding knowledge scores, past breastfeeding experience, a higher educational level, a full-time job, and female children [13,16].

Behavioral intention refers to an individual’s plan to engage in a certain behavior [17]. Women are more likely to exhibit behavioral intention to breastfeed if they have more motivation to breastfeed, positive attitudes toward breastfeeding, more breastfeeding self-efficacy, and higher levels of perceived benefits of breastfeeding [14,15].

Women who start breastfeeding their baby shortly postpartum, those who learn breastfeeding techniques, and those with high breastfeeding self-efficacy are more likely to successfully breastfeed their babies postpartum during the hospital stay [14,15]. Moreover, social attitudes toward breastfeeding, health care service quality, government policies, and legal concerns affect women’s intention to breastfeed their babies [18]. After discharge, health literacy, knowledge, intention, and self-efficacy were positively and significantly associated with breastfeeding [3]. According to the above literature review, we hypothesized that women who have insufficient knowledge of breastfeeding, a lack of behavioral intention to breastfeed, and less breastfeeding self-efficacy are less likely to breastfeed postpartum. In Taiwan, government policy has prohibited infant formula sales promotion since 1 January 2015, including the offering of gifts, samples, or discount coupons. Unfortunately, during their pregnancy period, women still acquire information regarding infant formula, which hampers their intention to breastfeed. The aims of this study were to (1) assess levels of breastfeeding knowledge, intention, and self-efficacy, and (2) identify the factors associated with breastfeeding during women’s postpartum hospital stays.

## 2. Materials and Methods

### 2.1. Design and Population

#### 2.1.1. Design

This longitudinal study employed a pretest and posttest design.

#### 2.1.2. Study Participants

The inclusion criteria were as follows: (1) age 20 years and above, (2) pregnancy between 30 and 34 gestational weeks with regular antepartum examinations at the outpatient department, (3) ability to communicate in Mandarin or Taiwanese, (4) ability to participate in this study and complete the questionnaires. Women were excluded if they met the following criteria: (1) having mental illness (according to a medical charts review), (2) being unstable or unable care for themselves, (3) having a visual or hearing impairment, and (4) having a high-risk pregnancy.

Those who delivered vaginally or through cesarean section stayed in hospital for 72 h and 120 h after postpartum, respectively. An amenity room was made available with the purpose of providing an environment (1) for mothers to breastfeed their babies and (2) for fathers to learn how to take care of the baby. If women felt unwell and preferred not to use the amenity room, their babies remained in the baby room.

#### 2.1.3. Setting

Participants were recruited using the consecutive sampling from March to November 2020. The women’s data were collected in the gynecology and obstetrics outpatient settings of a medical center in Taoyuan, Taiwan.

### 2.2. Data Collection

Pregnant women who met the inclusion criteria were contacted by a research nurse during their visits to the clinic in the hospital. Data were self-reported; participants who met the inclusion criteria fill structured questionnaires in a consultation room of the outpatient department. When necessary, the research nurse read each item of the questionnaire to the participant, which took approximately 10–15 min. Age, education level, employment status, parity type, prenatal health education level, and delivery method were obtained from the participants’ medical charts. The participants completed the questionnaires before they received antepartum examinations. The research nurse verified the content integrity of the questionnaire response.

### 2.3. Time Points of Data Collection

The women were assessed at two time points: pretest data were collected during the visit to the outpatient clinic at a gestational age between 30 and 34 weeks. Posttest data were collected postpartum during the hospital stay in the inpatient wards before discharge.

### 2.4. Ethical Considerations

The Ethical and Research Committee of Chang Gung Medical Foundation approved this study (number: 201601925B0C502). In this study, we followed ethical research principles by obtaining informed consent, ensuring confidentiality, and guaranteeing the right of withdrawal from research at any point of the study without any impact on the antepartum examinations or delivery procedures. We also informed participants that research nurses accessed the medical charts.

### 2.5. Variables of Interest

The study variables employed in this study are defined in the following sections.

#### 2.5.1. Exclusive Breastfeeding

Exclusive breastfeeding refers to the process of feeding only breast milk to an infant through the breast or bottle-feeding [19]. In this study, exclusive breastfeeding is defined as women only feeding their babies breast milk after delivery and until discharge.

#### 2.5.2. Breastfeeding Duration

Breastfeeding duration refers to the period between the initial breastfeeding until weaning [20]. In this study, breastfeeding duration is defined as the period starting from the women first feed breastfeeding her baby postpartum to hospital stay.

#### 2.5.3. Postpartum

Postpartum refers to the fourth stage of labor. The acute period involves the first 6–12 h postpartum, the subacute period refers to the 2–6 weeks postpartum, and the delayed period is the first 6 months after delivery [21]. In the current study, postpartum is defined as the period after delivery until hospital discharge.

### 2.6. Measurements

#### 2.6.1. Numeric Rating Scale

The Numeric Rating Scale (NRS) is a unidimensional measure of various problems [22,23]. The NRS is a horizontal bar or line with an 11-point scale from 0–10 (0 = no problem or intention at all, and 10 = potentially extreme problem or intention) [23]. In this study, participants were asked to rate their level of intention to breastfeed postpartum.

#### 2.6.2. Breastfeeding Knowledge Questionnaire

The Breastfeeding Knowledge Questionnaire (BKQ) was used to assess participants’ knowledge of breastfeeding [24]. This 26-item questionnaire has eight subscales: benefits (four items), component (two items), lactation mechanism (four items), skills (five items), breast problem management (three items), neonatal problem management (four items), contraindication (one item), and breast milk preservation (three items). Each correctly answered item was assigned a score of 1, and incorrectly answered items were assigned a score of 0. A previous study demonstrated that this scale has satisfactory psychometric properties [24]. Cronbach’s α for the BKQ used in this study was 0.80.

#### 2.6.3. Breastfeeding Self-Efficacy Scale—Short Form

Participants’ breastfeeding confidence was assessed using the Breastfeeding Self-Efficacy Scale—Short Form (BSES-SF) [25,26]. The instrument consists of 14 items, with responses scored on a Likert scale from 1 (not at all confident) to 5 (very confident), with higher scores indicating higher levels of breastfeeding self-efficacy. The reliability of the BSES-SF has been demonstrated previously for pregnant women [27,28]. In the present study, Cronbach’s α was 0.96.

#### 2.6.4. Demographic and Obstetrics Characteristics

A sheet of demographic and obstetrics characteristics was used to collect information, including age, education level, employment status, parity type, prenatal health education level, delivery mode, and breastfeeding status during the postpartum hospital stay period.

### 2.7. Statistical Analysis

Data were analyzed with SPSS (Statistical Package for the Social Sciences) version 26.0 for Windows (IBM Corp., Armonk, NY, USA). Data was examined, revealing a normal distribution; thus, parametric statistical analysis was used. Demographic characteristics, obstetrics characteristics, and participants’ breastfeeding intention, knowledge, and self-efficacy were analyzed using descriptive statistics (frequency distribution, percentage, means, and standard deviations (SDs)). Pearson’s product-moment coefficient was used to examine the relationships between breastfeeding status (dependent variable) and the selected independent variables (education level, knowledge, prenatal breastfeeding intention, self-efficacy, delivery mode (vaginal or cesarean)), and parity type (primiparous or multiparous)). A logistic regression analysis was used to identify factors associated with breastfeeding during the postpartum period in the hospital. The independent variables found to be statistically significant in the Pearson’s product-moment coefficient analysis were included in the logistic regression. Statistical significance was defined as *p* < 0.05.

## 3. Results

### 3.1. Demographic and Obstetrics Characteristics

Of the 125 eligible women who were invited to participate, five women declined to participate because of a lack of interest. The response rate was 96%.

The mean age of the participants was 33.21 (SD = 5.11) years, and the age range was 20 to 50 years. Nearly half of participants were educated at the university level (*n* = 59, 49.2%), reported being employed (*n* = 91, 75.8%), were primiparas (*n* = 67, 55.8%), had received prenatal health education (*n* = 97, 80.8%), and delivered vaginally (*n* = 86, 71.67%). After delivery, 25% of participants (*n* = 30) breastfed during the postpartum hospital stay (Table 1).

### 3.2. Scores for Breastfeeding Knowledge, Intention, and Self-Efficacy

The mean score for knowledge was 14.98 (SD = 4.43). The mean scores for the subscales of knowledge were as follows: benefits, 3.49 (SD = 0.76); component, 1.48 (SD = 0.75); lactation mechanism, 2.64 (SD = 0.91); skills, 2.03 (SD = 1.37); breast problem management, 1.71 (SD = 0.94); neonatal problem management, 1.11 (SD = 0.96); contraindication, 0.36 (SD = 0.48); and breast milk preservation, 2.15 (SD = 0.98). The mean antepartum score for intention to breastfeed was 56.29 (SD = 5.88). The mean score for breastfeeding self-efficacy was 41.55 (SD = 12.09) (Table 2).

### 3.3. BKQ Items with the Lowest Correct Response Rate

The knowledge items that were most frequently answered incorrectly were “Babies breastfeeding those whose defecation is loose” (*n* = 8, 6.7%), “If babies defecate eight times per day, this indicates that breastfeeding is sufficient” (*n* = 18, 15.0%), and “Mothers should hold the breast close to the nipple to help babies suck milk” (*n* = 21, 17.5%) (Table 3).

### 3.4. Correlates with Breastfeeding during the Postpartum Period

The factors that correlated significantly with breastfeeding during the postpartum period were education level (*r* = −0.22, *p* < 0.05), prenatal breastfeeding intention (*r* = 0.26, *p* < 0.01), self-efficacy (*r* = −0.20, *p* < 0.05), and parity type (*r* = −0.18, *p* < 0.05). These variables were included as independent variables in the logistic regression analysis (Table 4).

### 3.5. Factors Related to Breastfeeding during the Postpartum Period

A total of 30 (25%) participants reported breastfeeding during the postpartum hospital stay. Breastfeeding during the postpartum hospital stay period (yes or no) was used as the dependent variable in the logistic regression analysis, and the results indicated that a higher level of education and a higher level of prenatal breastfeeding intention were associated with breastfeeding during the postpartum hospital stay (Table 5).

### 3.6. Comparison of Demographic and Obstetrics Characteristics, Breastfeeding Intention, Knowledge, and Self-Efficacy between Women Who Did and Did Not Breastfeed Their Babies

Of the 120 women in this study, 30 women reported breastfeeding and 90 women reported not breastfeeding. The mean age of women did not differ significantly between these groups. Most women who breastfeed their babies had a lower educational level, were multiparous, and had higher levels of prenatal breastfeeding intention and breastfeeding self-efficacy; in these characteristics, significant differences were noted between these women and those who did not breastfeed. No further significant differences in patient characteristics were observed between the groups. The patient characteristics are summarized in Table 6.

## 4. Discussion

In this study, 25% of participants reported breastfeeding during the postpartum hospital stay, a much smaller percentage than that reported in previous studies [3,4]. A survey by Lee et al. [6] found that the rates of breastfeeding among women at 6 months postpartum in 2008, 2009, 2010, and 2011 were 32.2%, 41.4%, 27.3%, and 49.9%, respectively. Tsai et al. [3] surveyed 300 primiparous mothers in Taiwan, 66% of whom reported breastfeeding during the hospital stay; breastfeeding rates declined to 37.5% at 1 month and 30.2% at 3 months postpartum, and only 17.1% of women reported continuing breastfeeding at 6 months. These differences may have been influenced by employment status. In the present study, 75.8% (*n* = 91) of participants had full-time employment postpartum, whereas 60.8% of women reported full-time employment in the study by Tsai et al. [3] and 54.0% to 60.0% of women were employed outside the home in a study by Chiou et al. [4]. Although many workplaces have implemented baby-friendly practices, the creation of employer and coworker support and positive communication in the workplace may help women continue to breastfeed while working.

Participants in the present study were most likely to answer the following BKQ items incorrectly: “Mothers with diarrhea should not breastfeed their babies,” “If babies have eight bowel movements per day, this indicates that breastfeeding is sufficient,” and “Mothers with colds are unable to breastfeed.” These findings suggest that women lack knowledge regarding the breastfeeding of babies. Hence, in clinical settings, women should be provided with guidance on breastfeeding. For example, prenatal health education may include scenario-based simulation training related to breastfeeding [29]. Health care providers should assess women’s knowledge and provide breastfeeding practice to help women identify and prioritize breastfeeding problems, thereby reducing women’s barriers to breastfeeding and enhancing their skills during the early postpartum period.

The results of this current study showed that women who had higher prenatal levels of breastfeeding intention were more likely to breastfeed during their hospital stay. These findings are similar to those of Evans et al. [14], Park et al. [15], and Tsai et al. [3], who demonstrated that women with higher levels of breastfeeding self-efficacy were more likely to breastfeed. Providing sufficient information to assist women in transitioning to continued breastfeeding after hospital discharge may ease the breastfeeding strain. In clinical care, individual-oriented practice and learning for women are needed, including instruction in principles and skills of breastfeeding, early detection of problems, and resolution of common problems.

In Taiwan, most women stayed at a postpartum center after discharge. During the postpartum recuperation period, especially the early postpartum stage, women experience physical and psychological changes including pain, fatigue, and sleeping difficulties [30]; they also commonly feel overwhelming about caring for their baby [31]. During the first few weeks postpartum, women adjust to their new role and learn how to care for their baby. To foster successful breastfeeding, postpartum care should include the sharing of breastfeeding experience and instructions on self-care. Providers should be aware of women’ breastfeeding related concerns, particularly in the postpartum phase. Aromatherapy intervention could also be considered because it may improve postpartum physiological and psychological health, including anxiety, depression, distress, fatigue, post-recovery, sleep quality, and stress [31].

Results from the present study indicated that the level of education was negatively correlated with postpartum breastfeeding during the hospital stay. This finding conflicts with that of a previous study of national surveys in Taiwan, which indicated that education was positively associated with a greater likelihood of breastfeeding, especially among women with a university degree or higher education level [32]. Our own clinical observations indicate that women with higher educational levels are likely to be in full-time employment as midlevel manager or employed in other jobs with demanding schedules. This difference may be explained by differences in occupation, which can affect the ability of women to continue breastfeeding after returning to work. For example, women with higher education levels are more likely to have insufficient break time for expressing breast milk during working hours. Establishing a breastfeeding-friendly workplace environment may effectively increase rates of continued breastfeeding among working women. Thus, further research should address these issues.

This study has several limitations. First, we used a pretest and posttest design that assessed postpartum breastfeeding knowledge, intention, and self-efficacy during participants’ hospital stays. As breastfeeding after discharge is a long-term concern, a longitudinal investigation is necessary. Second, data collection for this study was based on convenience sampling. Participants were selected from a single outpatient ward of a medical center, which may limit the generalizability of the findings. Future research should expand sampling and compare postpartum breastfeeding knowledge, intention, and self-efficacy during hospital stays among women at multiple medical centers. Third, all of the instruments in this study were already tested and measured in previous gynecology- and obstetrics-related studies [24,25,26]. The current study involved women with a gestational age between 30 and 34 weeks who received regular antepartum examinations. Future studies should explore psychometric properties among women of different gestational ages. Finally, more family support and wider availability of resources in the workplace may promote breastfeeding continuation; however, we did not consider these factors. Comparative studies are necessary to determine the effect of supportive systems on postpartum breastfeeding among women.

## 5. Conclusions

### 5.1. Summary

A total of 120 participants were included in this study, 25% of whom reported breastfeeding during their hospital stay. Lower education levels and higher prenatal levels of breastfeeding intention were associated with higher rates of postpartum breastfeeding during the hospital stay.

### 5.2. Clinical Implications

Our study documents the factors associated with postpartum breastfeeding during the hospital stay. These findings provide a reference for the clinical assessment of continued breastfeeding and will help improve health care providers’ awareness of the significance of breastfeeding knowledge, intention, and self-efficacy.

## Figures and Tables

**Table 1:** Demographic and obstetrics characteristics (*N* = 120).

Variable	Number (%)	Mean (SD)	Range
Age (years)		33.21 (5.11)	20–50
Educational level			
Senior high	25 (20.8)		
College	9 (7.5)		
University	59 (49.2)		
Graduate	27 (22.5)		
Occupation			
Unemployed	29 (24.2)		
Employed	91 (75.8)		
Parity			
Primiparous	67 (55.8)		
Multiparous	53 (44.2)		
Prenatal health education			
No	23 (19.2)		
Yes	97 (80.8)		
Delivery mode			
Vaginal	86 (71.67)		
Cesarean	34 (28.33)		
Breastfeeding postpartum			
No	90 (75)		
Yes	30 (25)		

**Table 2:** Scores for breastfeeding intention, knowledge, and self-efficacy (*N* = 120).

Variable	Mean	SD	Range	Theoretical Scoring Range
Breastfeeding knowledge questionnaire (BKQ)	14.98	4.43	2–24	0–26
Benefits	3.49	0.76	1–4	0–4
Ingredient	1.48	0.75	0–2	0–2
Lactation mechanism	2.64	0.91	0–4	0–4
Skills	2.03	1.37	0–5	0–5
Breast problems management	1.71	0.94	0–3	0–3
Neonatal problems management	1.11	0.96	0–3	0–4
Contraindication	0.36	0.48	0–1	0–1
Breast milk preservation	2.15	0.98	0–3	0–3
Prenatal breastfeeding intention (NRS)	8.93	1.84	0–10	0–10
Self-efficacy (BSES-SF)	41.55	12.09	14–70	14–70

Abbreviations: NRS, Numeric Rating Scale, theoretical scoring range: 0–10. BKQ, Breastfeeding Knowledge Questionnaire, theoretical scoring range: 0–26. BSES-SF, Breastfeeding Self-Efficacy Scale—Short Form, theoretical scoring range: 14–70.

**Table 3:** BKQ items with the lowest rate of correct answers (*N* = 120).

Rank	Item Content	*N* (%)	Correct Answer Rate
1	12. Mothers with colds should not breastfeed.	56	46.7
2	9. Insufficient fluid intake leads to decreased breast milk production.	51	42.5
3	22. Continuous and light sucking by babies are effective behaviors.	50	41.7
4	10. Mothers should make a schedule for breastfeeding early in the postpartum period.	43	35.8
5	23. Mothers with hepatitis B are unable to breastfeed.	43	35.8
6	16. Mothers had inverted nipples cause babies to have difficulty sucking milk.	38	31.7
7	11. Hot compresses and breast massage before breastfeeding can promote the amount of breast milk.	24	20.0
8	15. Mothers should hold the breast close to the nipple to help babies suck milk.	21	17.5
9	19. If babies have eight bowel movements per day, this indicates that breastfeeding is sufficient.	18	15.0
10	21. Mothers with diarrhea should not breastfeed their babies.	8	6.7

**Table 4:** Correlations between breastfeeding during the postpartum period and selected independent variables (*N* = 120).

Variable	1	2	3	4	5	6	7
1. Education level (year)	1.00						
2. Knowledge	0.20 *	1.00					
3. Prenatal breastfeeding intention	0.20 *	0.20 *	1.00				
4. Self-efficacy	−0.16	0.06	0.24 **	1.00			
5. Delivery mode (vaginal vs. cesarean)	−0.10	0.05	−0.25 **	−0.08	1.00		
6. Parity (primiparous vs. multiparous)	−0.21 *	0.29 **	0.01	0.16	−0.01	1.00	
7. Breastfeeding status	−0.22 *	−0.02	0.26 **	−0.20 *	−0.07	−0.18 *	1.00

* *p* < 0.05; ** *p* < 0.01.

**Table 5:** Logistic regression analysis of factors related to breastfeeding during the postpartum period (*N* = 120).

Variable	Beta	SE	Wald Test	*p*	Odds Ratio (95% CI)
Educational level (year)	−0.290	0.116	6.218	0.013	0.595–0.940
Prenatal breastfeeding intention (NRS)	0.691	0.265	6.785	0.009	1.187–3.358
Self-efficacy (BSES-SF)	0.015	0.020	0.595	0.440	0.977–1.056
Parity (primiparous vs. multiparous)	0.559	0.475	1.386	0.239	0.690–4.436
Constant	−4.058	2.963	1.875	0.171	

Abbreviations: SE, standard error; CI, confidence interval. NRS, Numeric Rating Scale. BKQ, Breastfeeding Knowledge Questionnaire. BSES-SF, Breastfeeding Self-Efficacy Scale—Short Form. Chi-square = 111.729, *p* < 0.05, Nagelkerke R^2^ = 0.261. Input independent variable: covariates included education level (year) (continuous score), prenatal breastfeeding intention (continuous score), self-efficacy (continuous score), and parity type (primiparas vs. multiparas).

**Table 6:** Differences in demographic and obstetrics characteristics, breastfeeding intention, knowledge, and self-efficacy between the group that did and did not breastfeed their babies (*N* = 120).

Variable	Breastfeeding Group (*n* = 30)	Not Breastfeeding Group (*n* = 90)	*X*^2^/*t*	*p*
	*N* (%)/Mean (SD)	*N* (%)/Mean (SD)		
Age (years)	33.87 (6.84)	32.99 (6.84)	0.66	0.514
Educational level			10.81	0.013 *
Senior high	12 (40.0)	13 (14.4)		
College	1 (3.3)	8 (8.9)		
University	14 (46.7)	45 (50.0)		
Graduate	3 (10.0)	24 (26.7)		
Occupation			3.41	0.065
Unemployed	11 (36.7)	18 (20.0)		
Employed	19 (63.3)	72 (80.0)		
Parity			5.96	0.019 *
Primiparous	11 (36.7)	56 (62.2)		
Multiparous	19 (63.3)	34 (37.8)		
Prenatal health education			0.45	0.503
No	23 (76.7)	74 (82.2)		
Yes	7 (23.3)	16 (17.8)		
Delivery mode				
Vaginal	24 (80.0)	62 (68.9)	1.37	0.242
Cesarean	6 (20.0)	28 (31.1)		
Knowledge (BKQ)	15.07 (5.13)	15.16 (4.14)	0.10	0.918
Benefits	3.43 (0.82)	3.51 (0.74)		
Ingredient	1.33 (0.88)	1.53 (0.69)		
Lactation mechanism	2.63 (0.96)	2.64 (0.89)		
Skills	2.17 (1.53)	1.99 (1.32)		
Breast problems management	1.83 (0.95)	1.67 (0.94)		
Neonatal problems management	1.17 (1.12)	1.09 (0.91)		
Contraindication	0.30 (0.47)	0.38 (0.49)		
Breast milk preservation	2.20 (1.03)	2.13 (0.96)		
Prenatal breastfeeding intention (NRS)	9.80 (0.66)	8.63 (2.01)	4.78	0.001 **
Self-efficacy (BSES-SF)	46.47 (14.00)	40.06 (10.97)	2.58	0.011 *

Abbreviations: SD, standard deviation. * *p* < 0.05; ** *p* < 0.01.

## Data Availability

The data that support the findings of this study are available from the corresponding author. Restrictions apply to the availability of these data, which were used under licence for this study. Data are available from the authors with the permission of Chang Gung Memorial Hospital Research Program in Taiwan.
